# Cynomolgus macaques naturally infected with *Trypanosoma cruzi*-I exhibit an overall mixed pro-inflammatory/modulated cytokine signature characteristic of human Chagas disease

**DOI:** 10.1371/journal.pntd.0005233

**Published:** 2017-02-22

**Authors:** Danielle Marquete Vitelli-Avelar, Renato Sathler-Avelar, Armanda Moreira Mattoso-Barbosa, Nicolas Gouin, Marcelo Perdigão-de-Oliveira, Leydiane Valério-dos-Reis, Ronaldo Peres Costa, Silvana Maria Elói-Santos, Matheus de Souza Gomes, Laurence Rodrigues do Amaral, Andréa Teixeira-Carvalho, Olindo Assis Martins-Filho, Edward J. Dick, Gene B. Hubbard, Jane F. VandeBerg, John L. VandeBerg

**Affiliations:** 1 Grupo Integrado de Pesquisas em Biomarcadores, Centro de Pesquisas René Rachou, FIOCRUZ, Belo Horizonte, MG, Brazil; 2 Southwest National Primates Research Center, Texas Biomedical Research Institute, San Antonio, TX, United States of America; 3 Centro Universitário Newton Paiva, Belo Horizonte, MG, Brazil; 4 Faculdade de Minas–FAMINAS-BH, Belo Horizonte, MG, Brazil; 5 Universidad de La Serena, La Serena, Chile; 6 Departamento de Propedêutica Complementar, Faculdade de Medicina, Universidade Federal de Minas Gerais, Belo Horizonte, Minas Gerais, Brazil; 7 Laboratório de Bioinformática e Análise Molecular, Instituto de Genética e Bioquímica Universidade Federal de Uberlândia, Campus Patos de Minas, Patos de Minas, MG, Brazil; 8 Laboratório de Bioinformática e Análise Molecular, Faculdade de Ciência da Computação, Universidade Federal de Uberlândia, Campus Patos de Minas, Patos de Minas, MG, Brazil; 9 South Texas Diabetes and Obesity Institute, School of Medicine, the University of Texas Rio Grande Valley, Brownsville/Harlingen/Edinburg, TX, United States of America; Yeshiva University Albert Einstein College of Medicine, UNITED STATES

## Abstract

**Background:**

Non-human primates have been shown to be useful models for Chagas disease. We previously reported that natural *T*. *cruzi* infection of cynomolgus macaques triggers clinical features and immunophenotypic changes of peripheral blood leukocytes resembling those observed in human Chagas disease. In the present study, we further characterize the cytokine-mediated microenvironment to provide supportive evidence of the utility of cynomolgus macaques as a model for drug development for human Chagas disease.

**Methods and findings:**

In this cross-sectional study design, flow cytometry and systems biology approaches were used to characterize the *ex vivo* and *in vitro T*. *cruzi*-specific functional cytokine signature of circulating leukocytes from TcI-*T*. *cruzi* naturally infected cynomolgus macaques (CH). Results showed that CH presented an overall CD4^+^-derived IFN-γ pattern regulated by IL-10-derived from CD4^+^ T-cells and B-cells, contrasting with the baseline profile observed in non-infected hosts (NI). Homologous TcI-*T*. *cruzi*-antigen recall *in vitro* induced a broad pro-inflammatory cytokine response in CH, mediated by TNF from innate/adaptive cells, counterbalanced by monocyte/B-cell-derived IL-10. TcIV-antigen triggered a more selective cytokine signature mediated by NK and T-cell-derived IFN-γ with modest regulation by IL-10 from T-cells. While NI presented a cytokine network comprised of small number of neighborhood connections, CH displayed a complex cross-talk amongst network elements. Noteworthy, was the ability of TcI-antigen to drive a complex global pro-inflammatory network mediated by TNF and IFN-γ from NK-cells, CD4^+^ and CD8^+^ T-cells, regulated by IL-10^+^CD8^+^ T-cells, in contrast to the TcIV-antigens that trigger a modest network, with moderate connecting edges.

**Conclusions:**

Altogether, our findings demonstrated that CH present a pro-inflammatory/regulatory cytokine signature similar to that observed in human Chagas disease. These data bring additional insights that further validate these non-human primates as experimental models for Chagas disease.

## Author summary

*Trypanosoma cruzi* is the causative agent of Chagas disease; millions of people are infected with this parasite. One of the major challenges to manage infected patients is the low efficacy of currently available treatments, especially during chronic infection. Different *T*. *cruzi* genotypes are known to differ in response to existing drugs (e.g., TcI is quite resistant), and differences among individuals in immune response also are believed to play a role determining therapeutic efficacy. Experimental models and *in vitro* systems have been proposed for rational searches for new compounds for treating infected individuals, optimally before the infection becomes clinically manifested as Chagas disease. In the field of drug development, the non-human primate models offer a unique and valuable contribution, as a consequence of patho-physiological similarities that mimic many human diseases, including Chagas disease. In the present study, we further investigated the functional features of the immune response triggered by TcI *T*. *cruzi*-infection, characterizing the *ex vivo* as well as the *in vitro* cytokine microenvironment, upon *T*. *cruzi*-antigen recall. Our results revealed that chronically infected cynomolgus macaques display a similar *ex vivo* cytokine signature to that observed in chronic human Chagas disease. Moreover, CH macaques display a complex cross-talk among the cytokine^+^ leukocyte subsets, enhanced by TcI *T*. *cruzi*-antigen recall *in vitro*. These findings provide additional insights that further validate these non-human primates as experimental models for rational development of new therapeutic agents for Chagas disease.

## Introduction

Chagas disease, caused by the obligate intracellular protozoan *Trypanosoma cruzi*, is the most lethal endemic infectious disease and one of the most important public health problems in Latin America [1,2], where estimated 6 to 7 million people are infected and over 25 million are at potential risk [3]. Recent studies have reported the occurrence of Chagas disease in non-endemic areas of North America and Europe [4,5,6].

Major challenges to manage Chagas disease patients are the lack of immunoprophylactic tools and also the low efficacy of currently available compounds to treat Chagas disease, especially during chronic infection [7]. It has been extensively reported that the effectiveness of chemotherapeutic agents in treating Chagas disease is a multifactorial phenomenon influenced by parasite and host intrinsic features [7]. In this context, it is well known that the *T*. *cruzi* resistance to chemotherapeutic agents is associated with specific parasite genotypes, particularly TcI, that has been considered highly resistant to benznidazole and nifurtimox treatment [8]. In addition to the parasite genetic background, it has been extensively demonstrated that the host immune response plays an important role in determining the therapeutic effectiveness in Chagas disease [9].

Based on the low rates of parasitological cure reported for benznidazole and nifurtimox, the search for new drugs or the establishment of improved protocols for the etiological treatment of Chagas disease (including combined therapies) should be considered a priority to guide future perspectives for therapeutic management of Chagas disease patients. In this scenario, several scientific initiatives have been undertaken in an effort to meet the international demand for novel drugs in the near future.

In the field of drug discovery and development, *in vitro* anti-parasite screening combined with cell-line toxicity assays are relevant steps in identify promising compounds [10,11]. However, during pre-clinical research, pharmacokinetic studies involving absorption, distribution, metabolism, and excretion, together with acute toxicity evaluation in animal models, are required for regulatory approval of any drug intended for human use to enter clinical trials [12]. Non-human primate experimental models have been considered fundamental for research and development of medical products and devices since they reproduce most aspects of human illnesses and are the only models that manifest the complexity of the human physio-pathological conditions [13]. Moreover, non-human primates are the most suitable animal model because their immune system is very similar to that of humans. In fact, we have recently demonstrated that cynomolgus macaques naturally infected with *T*. *cruzi* present a range of *ex vivo* phenotypic features of circulating leukocytes that are highly similar to those observed in human Chagas disease patients [14]. We also have demonstrated that infected cynomolgus macaques with no macroscopic evidence of cardiac/digestive commitment have elevated levels of circulating monocyte-subsets, cytotoxic NK-cells and activated CD8^+^ T-lymphocytes, as is observed in human Chagas disease [14,15].

In the present study we have further evaluated the functional features of circulating leukocytes from cynomolgus macaques that had been naturally infected with *T*. *cruzi*, focusing on the *ex vivo* cytokine-mediated microenvironment and on *T*. *cruzi*-antigen recall *in vitro*. We have characterized the cytokine signatures of innate and adaptive immune compartments and applied system biology tools to provide information that further validates cynomolgus macaques as a highly appropriate primate model for pre-clinical research on Chagas disease.

## Materials and methods

### Study population

This is a cross-sectional investigation that enrolled 26 non-human primates (*Macaca fascicularis*), 21 females and five males, with ages ranging from 1–20 years and body weights ranging from 1.9 to 7.9 kg. Based on serological screening to detect anti-*T*. *cruzi* antibodies by enzyme-linked immunoassay (ELISA; Bio-Manguinhos; Oswaldo Cruz Foundation, Rio de Janeiro, Brazil) and immuno-chromatographic assay (Chagas STATPAK, Chembio Diagnostic Systems, Medford, NY), the monkeys were divided into two subgroups: *T*. *cruzi*-naturally infected macaques (CH), with positive serology in both tests, and non-infected controls (NI) displaying negative results in both tests. The CH group consisted of 15 non-human primates (12 females and three males) with median age of 12 years (age ranging from 2–20 years) and median weight of 3.5kg (weight ranging from 1.9–7.9 kg). All *T*. *cruzi*-naturally infected cynomolgus included in the present investigation presented the asymptomatic chronic phase of Chagas disease. This clinical form was determined by the absence of patent parasitemia and by meticulous organ inspections (esophagus, colon and heart) performed during necropsies. The myocardium of all monkeys showed a macroscopically normal aspect, without signs of wall aneurysms. Volume and weight of all hearts were within normal range. Moreover, the gastrointestinal tract did not present any macroscopic sign of megaesophagus or megacolon, suggestive of digestive chronic phase of Chagas disease. This approach followed the general criterion, according to Dias JC that macroscopically defines the indeterminate chronic Chagas disease [16]. The NI group included 11 animals (nine females and two males) with median age of 13 years (age ranging from 1–20 years) and median weight of 4.9kg (weight ranging from 1.9–7.6 kg).

### Ethics statement

The present investigation was approved by the Texas Biomedical Research Institute Animal Care and Use Committee (#1050MF) and was conducted in accordance with the Public Health Service Policy on Humane Care and Use of Laboratory Animals, and the U.S Animal Welfare Act. The macaques included in this study were breeders or their progeny and were housed in mixed sex social groups in metal/concrete indoor/outdoor enclosures at the Southwest National Primate Research Center (SNPRC) at the Texas Biomedical Research Institute, San Antonio, TX, USA. The indoor areas were heated during cool weather. The animals were provided commercial monkey chow and water *ad libitum*, supplemented with fruits and vegetables several times each week. Environmental enrichment devices, including balls and food puzzles, were provided at all times. Animal care was provided according to the Guide for the Care and Use of Laboratory Animals. No monkeys were euthanized for this investigation.

### Blood samples

Ten mL of heparinized whole blood samples were collected into Vacutainer tubes (BD Pharmingen, San Diego, CA, USA) from each animal. Peripheral blood was drawn from a femoral vein after immobilization of animals by general anesthesia with ketamine hydrochloride (10mg/kg) and valium (5mg) along with inhalation of isofluorane (1.5%).

### Molecular characterization of *T*. *cruzi* isolated from cynomolgus macaques

*T*. *cruzi* isolates were obtained from all seropositive macaques included in the present study by hemoculture as described previously by Luz et al. [17]. Epimastigote forms were harvested during Log-phase growth, washed three times with phosphate buffered saline–(PBS 0.015M, pH 7.4) at 1,000xg for 15min at 4°C, and the dry pellet stored at -70°C until processing. *T*. *cruzi* DNA was extracted as follows: aliquots containing 90μl of distilled water and 10μl of the parasites suspension were heated at 100°C for 10min and NanoDrop spectrophotometer (ThermoFisher Scientific, Waltham, MA, USA) was used to DNA quantification. Afterwards, the *T*. *cruzi* genomic DNA from each isolate were maintained at -20°C until use. Molecular characterization of *T*. *cruzi* isolates were performed using a combination of genetic markers, as follows: PCR-RFLP of the glucose-6-phosphate isomerase (GPI) locus using the BstEII enzyme and amplimer length variation of the 18S ribosomal RNA (rRNA) and mini-exon loci. For each locus, the PCR reactions were performed in 50μL reaction mixtures containing 100ng of *T*. *cruzi* genomic DNA, 1X DNA polymerase buffer (Ex Taq Takara for GPI; Amplitaq Gold Applied Biosystems for 18S rRNA and mini-exon), 2mM (18S rRNA and mini-exon) or 1.5mM (GPI) MgCl2, 200μM each dNTP, 500nM (GPI) or 1μM (18S rRNA and mini-exon) each primer, and 1U DNA polymerase (Ex Taq Takara for GPI; Amplitaq Gold Applied Biosystems for 18S rRNA and mini-exon). The GPI locus was amplified using the primer pairs and the cycling profiles described in Broutin et al. [18], and then digested with the BstEII restriction enzyme (NEB) according to manufacturer recommendations. The SNPRC TcIV type contains a cut site with this restriction enzyme at position 665 in the 1103bp amplified sequence (S1A Fig). For the 18S rRNA locus, primers described by Brisse et al. [19] were used. PCR was carried out by initial denaturation step of 10min at 94°C followed by 40 cycles of 30sec at 94°C, 30sec at 60°C, 30sec at 72°C, with a final extension step of 7min at 72°C (S1B Fig). For the mini-exon locus, we used the primers and multiplex approach described in Fernandes et al. [20], and a PCR program consisting of an initial denaturation step of 10min at 94°C followed by 40 cycles of 30sec at 94°C, 15sec at 54°C, 30sec at 72°C, with a final extension step of 7min at 72°C (S1C Fig). The amplified/digested products were detected by electrophoresis on 1.5% (GPI) or 2.5% (18S rRNA and mini-exon) agarose gels in TBE 0.5X (45mM Tris-borate, 1mM EDTA) and staining with ethidium bromide. Reference TcI and TcIV *T*. *cruzi* strains isolated from infected baboons from SNPRC and negative controls were included on each PCR batch.

### TcI and TcIV *T*. *cruzi* antigen preparations

The TcI and TcIV *T*. *cruzi* strains used in this study were obtained from naturally infected baboons (*Papio hamadryas*) maintained at SNPRC. The TcI and TcIV *T*. *cruzi* epimastigote organisms were isolated by hemocultures according to Luz et al. [17]. Epimastigote forms were harvested at the stationary growth phase in LIT (Liver Infusion Tryptose) culture medium and the viability tested by trypan blue-exclusion staining under light microscopy. Epimastigote forms were washed twice with PBS at 800xg, for 10 min at 4°C and the parasites suspension was adjusted to 1x10^8^ cells/mL. Parasites were stored as pellets at -80°C until use. The soluble *T*. *cruzi* antigens were prepared by three thawing/freezing steps (37°C for 10 min and -80°C for 2 min) followed by a 30 sec sonication process in a tissue homogenizer with a teflon piston (The VirTis Company, Gardiner NY, USA). The parasite lysates were centrifugated at 37.000xg for 90 min at 4°C to obtain the soluble fractions. The supernatant was collected, dialyzed against PBS for 72 h at 4°C, and filtered through 0,22μm membranes (Filter milex HA, USA): the resulting protein concentration was determined by the Lowry method. The soluble TcI and TcIV *T*. *cruzi* antigens were stored in 250μg/mL aliquots at -80°C until use for short-term whole blood cultures *in vitro*.

### Short-term whole blood cultures *in vitro*

Short-term whole blood cultures *in vitro* were performed as previously described by Vitelli-Avelar et al. [15]. Briefly, two distinct culture platforms referred to as: “control” and “antigen-stimulated” cultures were carried out in parallel for each blood sample.

Triplicates of “control” cultures were performed in 14mL polypropylene tubes (BD Pharmingen, San Diego, CA, USA), using 500μL aliquots of heparinized whole blood samples, incubated in the presence of 500μL of RPMI-1640 (GIBCO, Grand Island, NY, USA) plus Brefeldin A (BFA) (Sigma, St Louis, MO, USA) at a final concentration of 10μg/mL for 4 h at 37°C in a 5% CO_2_ humidified incubator. The “control” culture condition was referred to as “*ex vivo*”, which is expected to reflect the dynamic of events taking place *in vivo*, particularly in the absence of exogenous stimuli.

Triplicates of “antigen-stimulated” cultures were also performed in 14mL polypropylene tubes (BD Pharmingen, San Jose, CA, USA) and consisted of pre-incubation of 500μL aliquots of heparinized whole blood samples in the presence of 400μL of RPMI-1640 (GIBCO, Grand Island, NY, USA) plus 100μL of TcI or TcIV *T*. *cruzi* soluble antigens preparations at a final concentration of 20μg/mL, for 1 h at 37°C in a 5% CO_2_ humidified incubator. Following *T*. *cruzi* antigen recall *in vitro*, samples were re-incubated in the presence of BFA at 10μg/mL for an additional period of 4 h at 37°C in a 5% CO_2_ humidified incubator.

Following the short-term stimulation *in vitro*, all the cultures were treated with 100μL of 20mM EDTA final concentration (Sigma, St Louis, MO, USA), followed by incubation at room temperature for 10 min to stop the activation process.

The triplicates of each culture condition were pooled together prior to immunostaining for intracellular cytokine analysis by flow cytometry.

### Immunostaining for intracellular cytokine analysis by flow cytometry

After short-term culture *in vitro*, whole blood samples were immunostained as previously described by Vitelli-Avelar et al. [15]. Briefly, cultured samples were washed once with FACS buffer—PBS 0.5% of bovine serum albumin and 0.1% sodium azide (Sigma, St Louis, MO, USA) by centrifugation at 400xg, for 10 min at 4°C. Following, the cells were re-suspended 1.5mL of FACS buffer and 100μL aliquots were transferred to 5mL polystyrene tubes (BD Pharmingen, San Jose, CA, USA) containing FITC-labeled (BD Pharmingen, San Diego, CA, USA) or TC-labeled (Caltag, Burlingame, CA, USA) anti- CD14, HLA-DR, CD16, CD3, CD4, CD8 or CD20 mAbs. Aliquots were incubated 30 min at room temperature, in the dark. Following incubation, erythrocytes were lysed and leukocytes were fixed using 2mL of FACS lysing solution (BD Pharmingen, San Diego, CA, USA) for 20 min at room temperature, in the dark. The fixed membrane-stained leukocytes were then permeabilized with FACS perm-buffer, (FACS buffer supplemented with 0.5% of saponin purchased from Sigma, St Louis, MO, USA) by incubation for 30 min at room temperature, in the dark. Following permeabilization, the cells were stained with PE-labeled anti-cytokine (TNF, IFN-γ and IL-10) mAbs and incubated for 30 min at room temperature, in the dark. After intracytoplasmic cytokine staining, the cells were washed once with FACS perm-buffer and once with FACS buffer and then fixed in 200μL of FACS FIX Solution (10g/L paraformaldehyde, 10.2g/L sodium cacodylate, 6.65g/L sodium chloride, pH 7.2). The fixed cells were stored at 4°C up to 24 h until flow cytometry was conducted. A total of 30,000 events per sample were run in a CyAn ADP flow cytometry analyzer (Beckman Coulter, Inc., Brea, CA, USA). The Summit software 4.3.01 (Beckman Coulter, Inc., Brea, CA, USA) was used for data acquisition and storage. The FlowJo software (version 9.4.1, TreeStar Inc. Ashland, OR, USA) was applied for data analysis, using distinct gating strategies, as proposed by Vitelli-Avelar et al. [15]. The results were obtained as the percentage of cytokine^+^ cells (TNF^+^, IFN-γ^+^ and IL-10^+^) within a given leukocyte subpopulation (CD14^+^, CD14^+^HLA-DR^++^, CD16^+^, CD3^+^, CD4^+^, CD8^+^ or CD20^+^).

### Data analysis

#### Comparative analysis of cytokine-producing cells

The percentage of cytokine^+^ cells from innate and adaptive immunity was compared by Student’s t-test to identify significant differences between *T*. *cruzi*-naturally infected hosts and non-infected controls at p<0.05. The GraphPad Prism software (version 5.03, San Diego, California, USA) was used for statistical analysis, scattering plots assembling and final graphical art.

#### Frequency of high cytokine producers

The frequency of high cytokine producers was calculated as previously proposed by Luiza-Silva et al. [21]. Briefly, the global median value of cytokine^+^ cells was calculated for each leukocyte subpopulation, using the entire data universe (NI+CH, n = 26 monkeys) and used to categorize each animal as a “low” (below or equal to the global median) or “high” (higher then global median) cytokine producer. Diagrams were used to compile the number of high cytokine producers for each group and column statistics were run to quantify the percentage of animals classified as high producers within NI and CH. Microsoft Excel Software was used for diagram compilation and column statistics.

#### Establishing the overall cytokine signatures

The use of this approach was adapted from a pioneering study published by Luiza-Silva et al. [22], which allows the identification of subtle differences usually not detectable by conventional statistical approaches, but relevant to understanding patho-physiologic microenvironments. For this purpose, initially, the percentage of high producers calculated for each cytokine^+^ leukocyte subset was compiled on radar charts to create the overall cytokine signatures. Thereafter, the members of the cytokine^+^ leukocyte subset with frequencies above the 50^th^ percentile were highlighted and considered to be a relevant biomarker to interpret the overall cytokine signature. The relevant biomarkers are underscored by bold underline formats in all figures. Microsoft Excel Software was used for radar chart arts and the GraphPad Prism software (version 5.03, San Diego, California, USA) was used for bar charts and continuous line assembling and graphic art.

#### Ascendant cytokine signatures

Ascendant cytokine signatures curves were assembled to identify the leukocyte subset with the most prominent contribution for the high cytokine producers, as previously proposed by Campi-Azevedo et al. [22]. The biomarkers with frequencies above the 50^th^ percentile were considered relevant results and were highlighted by bold underline format or tagged by rectangles to highlight the pro-inflammatory (black rectangle) and modulatory (gray rectangle) cytokine-producing leukocyte subset. The GraphPad Prism software (version 5.03, San Diego, California, USA) was used for bar charts and continuous line assembling, overlaying plots and graphic art.

#### Biomarker network analyses

Systems biology tools were applied to construct biomarker networks and to assess the association between cytokine^+^ leukocyte subsets, their cross-talk and neighborhood connections for each clinical group. Conventional statistical analysis was performed by Spearman’s correlation test using the GraphPad Prism software (version 5.03, San Diego, California, USA). A matrix composed of all cytokine^+^ leukocyte subsets evaluated in this study was created and column statistics were applied to identify the significant correlations at p<0.05. Correlation matrices for cytokine^+^ cells were built using Microsoft Excel Software and the file was imported to the open source software Cytoscape (version 3.1.1) to create circular layouts biomarker networks as previously reported Shannon et al. [23]. Biomarker networks are displayed by node sizes reflecting the number of neighborhood connections from 0 to 8, according to the scale provided in the figure. Black and gray nodes were used to identify pro-inflammatory (TNF and IFN-γ) and regulatory (IL-10) cytokines respectively. Connecting edges are displayed with distinct thickness, representing the correlation scores, categorized as strong positive (r ≥ 0.68; thick black line), moderate positive (0.36 ≤ r < 0.68; thin black line), strong negative (r ≤ -0.68; thick gray dashed line), moderate negative (-0.68 < r ≤-0.36; thin gray equal dashed line) as proposed by Taylor [24].

## Results

### *Ex vivo* functional profile of peripheral blood leukocytes

**Fig 1:**
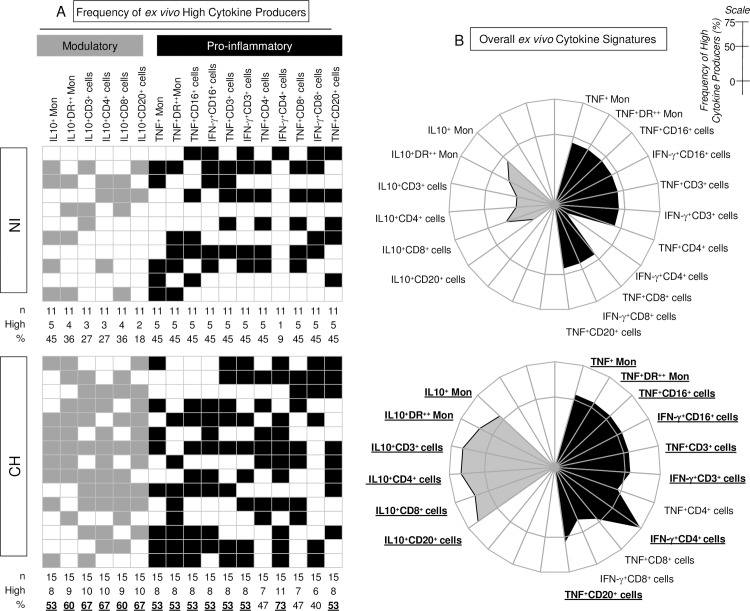
*Ex vivo* frequency of high cytokine producers and overall signatures in macaques naturally infected with TcI *T*. *cruzi*. **(**A) Gray-scale diagrams were used to compile the *ex vivo* frequency of high modulatory (gray square) and pro-inflammatory (black square) cytokine producers within *T*. *cruzi*-infected cynomolgus macaques (CH) and non-infected controls (NI). The global median values of cytokine^+^ cells were calculated, taken from the whole data universe (NI+CH, n = 26 non-human primates) and used as the cut-off mark to categorize each animal as “low” (white square) or “high” (gray square, black square) cytokine producers among NI and CH. Column statistics were run to quantify the frequency of high producers in each group. The biomarkers with frequencies above the 50^th^ percentile are highlighted by bold underline format. (B) Radar charts summarizing the modulatory (gray area) and pro-inflammatory (black area) cytokine signatures in a range of leukocyte subsets (monocytes, NK-cells, T-cells and B-cells) were plotted to evaluate the proportion of high producers within a given cell subpopulation. The frequencies of high producers confined outside the inner circle (50^th^ percentile) are underscored by bold underline format.

The frequency of *ex vivo* high cytokine producers and the overall *ex vivo* cytokine signatures of healthy and monkeys naturally infected with TcI *T*. *cruzi* are shown in Fig 1. Data analyses demonstrated that while non-infected primates displayed a baseline cytokine signature, infected macaques displayed an overall pro-inflammatory response with prominent levels of CD4^+^-derived IFN-γ, regulated by IL-10 which is produced by T-cells (CD4^+^ and CD8^+^) and B-cells (Fig 1).

**Fig 2:**
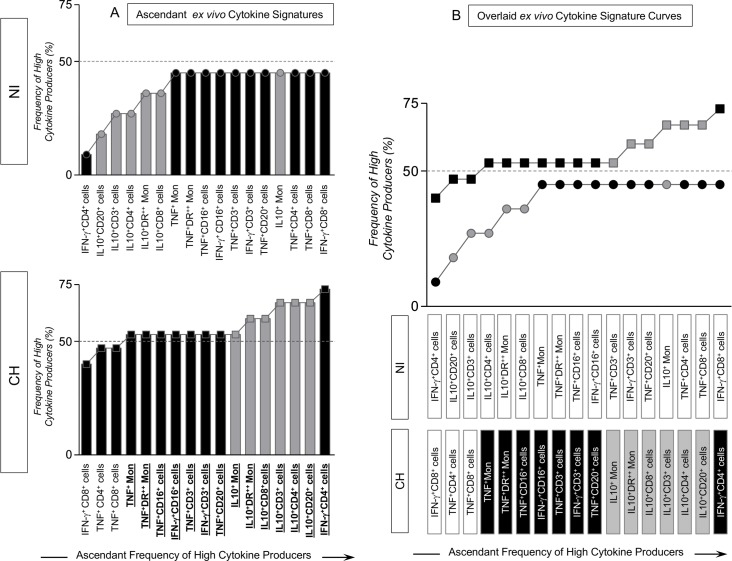
Ascendant *ex-vivo* cytokine signatures and overlaid comparative analyses of macaques naturally infected with TcI *T*. *cruzi* and non-infected macaques. (A) Ascendant *ex vivo* cytokine signatures assembled to characterize the frequency of high producers within infected monkeys (CH) and non-infected controls (NI). Data are presented by bar charts and continuous lines (NI = circle symbols; CH = Square symbols). The biomarkers with frequencies above the 50^th^ percentile are highlighted by bold underline format and the ascendant construction was used to identify the biomarkers with the most prominent contribution for high cytokine producers. (B) Overlaid cytokine signature curves were plotted for comparative analyses of the overall *ex vivo* ascendant cytokine pattern of CH and NI. The frequencies of high producers confined above the 50^th^ percentile line were tagged by rectangles to highlight the pro-inflammatory (black rectangle) and modulatory (gray rectangle) cytokine-producing leukocyte subset.

Noteworthy was the shift of IFN-γ^+^CD4^+^ cells from the lowest position in the cytokine signature of non-infected monkeys to the highest point in the ascendant cytokine signature of CH hosts. It is important to highlight that monkeys naturally infected with TcI *T*. *cruzi* also displayed a prominent *ex vivo* regulatory pattern mediated by IL-10 derived from monocytes, and T- and B-cells (Fig 2A). Additional analyses of overlaid ascendant *ex-vivo* cytokine signatures (Fig 2B) demonstrate that the frequency of high cytokine producers among non-infected monkeys are all confined below the 50^th^ percentile cut-off point. Remarkably, the CH hosts presented high frequencies of pro-inflammatory cytokine producers, including TNF from monocytes, NK-cells, T-cells and B-cells and IFN-γ from NK-cells and T-cells (Fig 2B).

Data analyses performed using the original dataset, expressed as percentage cytokine^+^ cells from innate and adaptive immunity, confirmed all significances mentioned above (S2 Fig).

### Impact of TcI and TcIV *T*. *cruzi* antigen priming/recall *in vitro* on the overall cytokine signature of peripheral blood leukocytes

**Fig 3:**
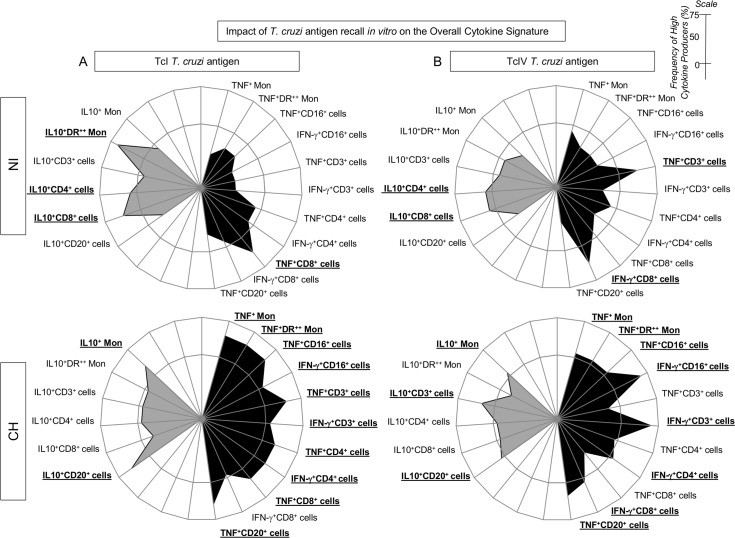
Overall cytokine signatures of *T*. *cruzi*-infected and non-infected macaques upon (TcI/TcIV)/*T*. *cruzi*-antigen recall *in vitro*. Radar charts were plotted to characterize the impact of (A) TcI and (B) TcIV *T*. *cruzi* antigen recall *in vitro* on the pro-inflammatory (black area) and modulatory (gray area) cytokine pattern of naturally infected cynomolgus macaques (CH) and non-infected controls (NI). The frequencies of high cytokine producers confined outside the inner circle (50^th^ percentile) are underscored by bold underline format.

The analyses of *in vitro* overall cytokine signature of peripheral blood leukocytes from healthy primates upon *T*. *cruzi* antigen priming/recall *in vitro* are shown in Fig 3A (TcI antigen) and Fig 3B (TcIV antigen). Data analyses demonstrated that the TcI antigen-priming *in vitro* was able to trigger, in non-infected monkeys, a modest pro-inflammatory response provided by TNF^+^CD8^+^ T-cells along with a prominent IL-10-modulated cytokine microenvironment supported by HLA-DR^++^ monocytes and T-cells (Fig 3A). On the other hand, the TcIV antigen-priming *in vitro* induce a prominent pro-inflammatory response characterized by enhanced frequency of TNF^+^ T-cells and IFN-γ^+^ CD8^+^ cells with minor IL-10 production derived from CD4^+^ and CD8^+^ T-cells (Fig 3B).

The impact of *T*. *cruzi* antigen-recall *in vitro* overall cytokine signature of peripheral blood leukocytes from CH is shown in Fig 3A (TcI) and 3B (TcIV). The results demonstrate that the homologous *T*. *cruzi* antigen (TcI) triggers a broad pro-inflammatory response supported by TNF and IFN-γ from innate/adaptive immunity cells counterbalanced by IL-10 produced by monocytes and B-cells (Fig 3A). Conversely the heterologous TcIV antigen induced a slighter pro-inflammatory response provided by TNF from monocytes, NK-cells and B-cells together with IFN-γ from NK-cells and T-cells, modulated by IL-10 derived from monocytes, T and B-cells (Fig 3B).

### Changes on the ascendant cytokine signature pattern of peripheral blood leukocytes elicited by TcI and TcIV *T*. *cruzi* antigen priming/recall *in vitro*

**Fig 4:**
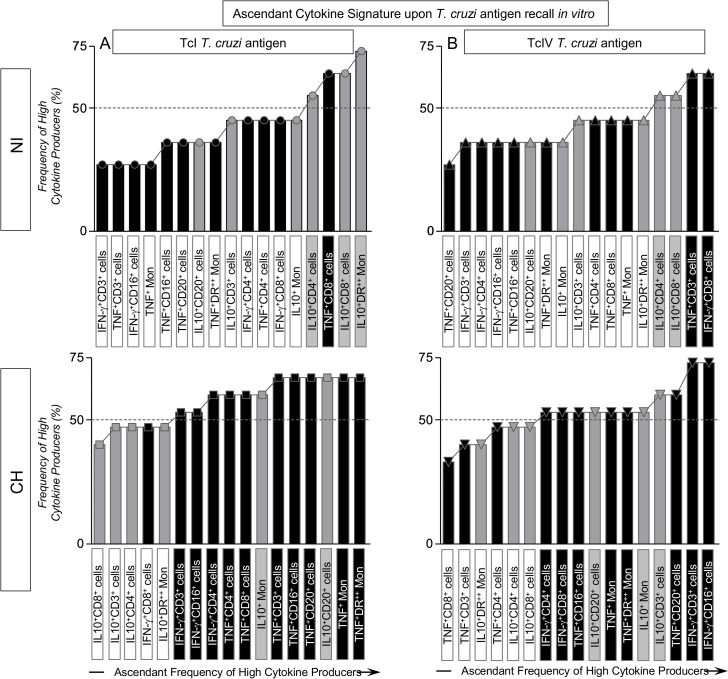
Ascendant cytokine signatures of *T*. *cruzi*-infected and non-infected macaques upon (TcI/TcIV)/*T*. *cruzi*-antigen recall *in vitro*. Bar charts and continuous lines were assembled together to characterize the differential impact of (A) TcI (NI = circle symbols; CH = Square symbols) and (B) TcIV (NI = triangle symbols; CH = inverted triangle symbols) *T*. *cruzi* antigen recall *in vitro* on the pro-inflammatory (black rectangle) and modulatory (gray rectangle) cytokine pattern of cynomolgus macaques naturally infected with *T*. *cruzi* (CH) and non-infected controls (NI). The ascendant constructions were used to identify the biomarkers with the most prominent contribution for high cytokine producers. The biomarkers with frequencies above the 50^th^ percentile line were tagged by rectangles to highlight the pro-inflammatory (black rectangle) and modulatory (gray rectangle) cytokine-producing leukocyte subset.

The analysis of ascendant cytokine signatures upon antigen recall represents a useful tool to identify functional biomarkers that can be applied to characterize microenvironment mediating the *T*. *cruzi*-specific immune response. Fig 4 shows the ascendant signature of circulating monocytes and lymphocytes from CH in response to homologous (Fig 4A) and heterologous (Fig 4B) *T*. *cruzi* antigens as well as the cytokine pattern of NI controls (Fig 4A and 4B). Results showed that TcI antigens trigger a predominantly regulatory cytokine profile in NI hosts, mainly orchestrated by IL-10 from T-cells and monocytes with minor TNF production by CD8^+^ T-cells (Fig 4A). On the other hand, TcIV antigen trigger a robust pro-inflammatory response in NI hosts mediated by TNF and IFN-γ produced by T-cells counterbalanced by IL-10 derived from CD4^+^ and CD8^+^ T-cells (Fig 4B).

It was noteworthy that Chagas disease hosts develop, upon homologous *T*. *cruzi* antigen recall *in vitro*, a prominent pro-inflammatory response involving IFN-γ (NK-cells and T-cells) together with TNF (monocytes, NK-cells, T-cells and B-cells) modulated by IL-10 from monocytes and B-cells (Fig 4A). In an opposite way, the heterologous *T*. *cruzi* antigens triggered a moderate cytokine pattern with modest frequencies of high cytokine producers (Fig 4B).

### Cytokine networks connecting peripheral blood leukocytes upon TcI and TcIV *T*. *cruzi* antigen priming/recall *in vitro*

**Fig 5:**
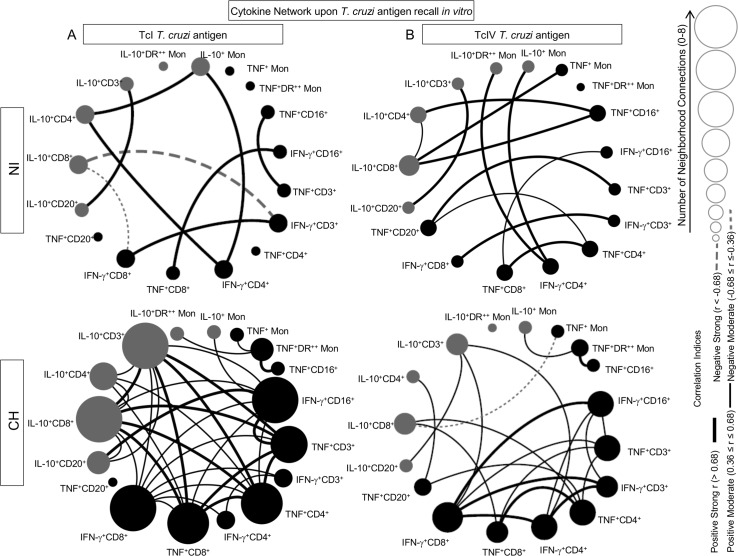
Systems biology analysis of cytokine network upon (TcI/TcIV)/*T*. *cruzi* antigen recall *in vitro*. Correlation matrices for cytokine^+^ cells were built with significant indexes and circular layouts to characterize the differential impact of (A) TcI and (B) TcIV *T*. *cruzi* antigen recall *in vitro*. Biomarker networks for cynomolgus macaques naturally infected with *T*. *cruzi* (CH) and non-infected controls (NI) are displayed by clustered distribution of nodes for pro-inflammatory (black node) and modulatory (gray node) cytokine patterns. Biomarker networks are displayed by node sizes reflecting the number of neighborhood connections from 0 to 8, according to the scale provided in the figure. Significant correlations (p≤0.05) were represented by connecting edges to underscore strong positive (r > 0.68; thick black line), moderate positive (0.36 ≤ r ≤ 0.68; thin black line), strong negative (r < -0.68; thick gray dashed line), moderate negative (-0.68 ≤ r ≤-0.36; thin gray equal dashed line), as proposed by Taylor [23].

Systems biology approaches were applied to construct cytokine networks based on the overall correlation between cytokine producing cells in order to better understand the functional properties and cross-talk among circulating leukocytes in CH primates. Correlation indexes and neighborhood connections were considered to assemble circular network layouts presented in Fig 5. Regardless of the *T*. *cruzi* genotype used for antigen priming *in vitro*, NI primates presented a cytokine network comprised by small number of neighborhood connections with strong correlation edges (Fig 5A Fig 5B). Remarkably, CH hosts displayed a more complex cross-talk amongst network elements, with enlarged nodes representing higher number of neighborhood connections (Fig 5A). Noteworthy was the ability of homologous *T*. *cruzi* antigen-recall to drive an outstanding pro-inflammatory response strongly linked to IL-10, whereas the heterologous antigen triggered a more modest network with lower number of edges (Fig 5B).

## Discussion

In this study we presented additional evidence that cynomolgus macaques, naturally infected with *T*. *cruzi* exhibit an overall immune response resembling that observed in human Chagas disease, characterized by a mixed pro-inflammatory/modulated cytokine signature. These findings further validate this monkey species as a valuable primate model for pre-clinical research on drug development for human Chagas disease. It is well known that the diversity of *T*. *cruzi* genotypes are not only associated with distinct patterns of Chagas disease morbidity but also with extent of drug resistance and susceptibility, where TcI strains are particularly resistant to chemotherapy by comparison with TcII [25,26,27]. Moreover, the parasite-host interfaces, especially the antigen-specific immune response, play a role determining drug activity of distinct compounds against *T*. *cruzi* organisms. In this sense, it is mandatory that *in vivo* studies, describing immunomodulatory effects of *in silico*/*in vitro* pre-selected hits, be carried out during pre-clinical research prior to approval of clinical trials or any drug intended for human use [12].

The murine experimental models for *T*. *cruzi* infection are usually employed in the field of drug discovery and development, mainly due to relative low cost, easy handling procedures, and rapid data output. Moreover, the availability of standardized protocols for post-therapeutic cure assessment, including protocols involving immunosuppression, contribute to the choice of mice to serve as the frontline models for drug discovery for Chagas disease [28].

However, one of the major concerns of using murine models as an isolated approach during pre-clinical research is that they do not reproduce several aspects of human illnesses, neither the precise physio-pathological features nor the complexity of the immune response. Therefore, non-human primate experimental models have been considered fundamental for pre-clinical research and development of medical products and devices [13]. We have previously reported that cynomolgus macaques naturally infected with *T*. *cruzi* display a phenotypic profile of circulating leukocytes similar to that observed in human Chagas disease. In the present study, we further characterized the functional aspects of circulating leukocytes from cynomolgus macaques using *ex vivo* and *in vitro* perspectives that simulate the parasite-host interface in three distinct situations, including: i) the first parasite-host contact, ii) the consolidated immunological events during chronic infection, and iii) the putative immune response triggered by *T*. *cruzi* antigen-recall, resembling endogenous antigen release.

Deciphering the differential impact of TcI and TcIV antigen priming *in vitro* on circulating leukocytes from non-infected monkeys, we intended to provide insights pertinent to the higher prevalence of TcI natural infection observed among the non-human primates at the SNPRC. Noteworthy was the distinct immunological profile observed for NI monkeys upon TcI and TcIV antigenic stimulation *in vitro*. While TcIV induced a prominent pro-inflammatory pattern mediated by TNF^+^ T-cells and IFN-γ^+^ CD8^+^ cells with minor IL-10 production derived from CD4^+^ and CD8^+^ T-cells, TcI antigen-priming was able to trigger a prominent IL-10-modulated cytokine microenvironment supported by HLA-DR^++^ monocytes and T-cells with a modest pro-inflammatory response provided by TNF^+^CD8^+^ T-cells. These findings support the general hypothesis that the strong pro-inflammatory response mounted by NI animals upon TcIV priming *in vitro* may play a protective role against this *T*. *cruzi* strain. The relevance of TNF as a protection-related biomarker has already been demonstrated; it elicits trypanocidal events mediated by activated macrophages and enhanced nitric oxide production [29]. Moreover, the higher frequency of IFN-γ^+^ T-cells with minor IL-10 also represents an additional trypanocidal pathway that contributes for TcIV clearance. On the other hand, the IL-10-modulated pattern elicited by TcI-derived antigen provides evidence of susceptibility to infection with this genotype. These proposals agree with the fact that TcI infection was predominant amongst the cynomolgus macaques, regardless of the fact that both genotypes are present among these and other non-human primates at the SNPRC.

Characterizing the *ex vivo* cytokine pattern of CH group, we observed an overall mixed pro-inflammatory/regulatory cytokine signature, mediated by IFN-γ from CD4^+^ T-cells counterbalanced by IL-10 produced by CD4^+^ T-cells and B-cells. This microenvironment resembles that previously described for chronic Chagas disease in humans. There are several lines of evidence that IFN-γ plays a dual role during *T*. *cruzi* infection, being involved in both protective and deleterious immune responses. Although, the IFN-γ production by NK-cells and T-lymphocytes is crucial for parasite clearance, activating macrophages and cytotoxic T-cells, it can also lead to tissue damage if an uncontrolled pro-inflammatory response takes place during chronic infection [30]. Therefore, the establishment of an IL-10-modulated immune response plays an essential role in containing any putative deleterious pro-inflammatory response [31,32]. Previous studies have investigated the cytokine profile triggered during the course of *T*. *cruzi* infections in *Cebus apella* monkeys [33]. It has been observed that chronically infected *C*. *paella* expressed a non-polarized immune response in the cardiac tissue, characterized by elevated levels IL-1β, IL-6, TNF-α along with TGF-β and IL-10.

Considering that the *in vitro* TcI *T*. *cruzi*-antigen recall approach to simulating the endogenous antigen release resultant of both parasite killing during chronic infection or massive parasite destruction triggered by therapeutic intervention, we observed a balanced pro-inflammatory/regulatory cytokine signature. This mixed microenvironment was created by TNF and IFN-γ derived from innate/adaptive cells, counterbalanced by monocytes/B-cells/CD8^+^ T-cells-derived IL-10. As mentioned above, the establishment of a balanced TNF/IFN-γ/IL-10 pattern is critical to control parasite growth with simultaneous maintenance of tissue integrity around inflammatory sites during chronic infection [30,34]. Moreover, the data regarding TcI-antigen recall *in vitro* further contributes to understanding the immunological events that may be elicited upon the massive endogenous antigen release that occurs during therapeutic drug treatment. There is evidence that an immune response mediated by high levels of IFN-γ acts synergistically with the drug treatment on trypanocidal events [31,35]. In fact, Sathler-Avelar et al. [31,36,37] suggested that the establishment of a type 1-modulated immune profile, with IFN-γ-mediated pro-inflammatory response together with high levels of IL-10 are key elements for parasite clearance in the absence of deleterious tissue damage that eventually might occur during Chagas disease treatment. These data reinforce the notion that non-human primates naturally infected with *T*. *cruzi* serve as an excellent model to test the hypothesis that the host immune response plays a synergistic role with therapeutic drugs in determining efficacy of treatment.

In summary, our data showed that monkeys naturally infected with TcI presented a mixed pro-inflammatory/regulatory response which resembled the immunological findings observed in humans Chagas disease. Taking into consideration the natural resistance of TcI to currently available drugs and the physiological and immunological similarities that non-human primates share with humans, our results further support the use of these animals as an experimental model that is uniquely suited for research on Chagas disease.

## Supporting information

S1 Checklist STROBE Checklist. The Checklist for a cross-sectional study was fulfilled and added in the online submission system as Vitelli-Avelar et al STROBE checklist file.
(PDF)

S1 Fig Molecular characterization of *T*. *cruzi* isolates from Cynomolgus macaques. (A) PCR-RFLP characterization of the *T*. *cruzi* isolates from seropositive cynomolgus macaques (CH20857, CH20575, CH21268, CH20567, CH20445, CH20657, CH20476, CH20281, CH21221 and CH20443) using the GPI locus digested with the BstEII restriction enzyme as described in Material and Methods. (B) Characterization of the *T*. *cruzi* isolates from seropositive cynomolgus macaques (CH20857, CH20575, CH21268, CH20567, CH20445, CH20657, CH20476, CH20281, CH21221 and CH20443) using length variation at the 18S ribosomal RNA locus. (C) Characterization of the *T*. *cruzi* isolates from seropositive cynomolgus macaques (CH20857, CH20575, CH21268, CH20567, CH20445, CH20657, CH20476, CH20281, CH21221, CH20443, CH20564, CH21395, CH20805 and CH20787) using the combination of mini-exon primers Tc1, Tc3 and ME, previously described by Fernandes et al. [20]. Reference TcI and TcIV *T*. *cruzi* strains isolated from infected baboons from SNPRC and negative controls were included in the PCR batches.
(TIF)

S2 Fig *Ex vivo* profile of cytokine-producing cells from innate and adaptive immunity in TcI *T*. *cruzi*-naturally infected macaques. (A) Pro-inflammatory and (B) modulatory cytokine-producing cells in Chagas disease Cynomolgus hosts (CH, black circles) and non-infected controls (NI, white circles). The results are expressed as mean values and scattering distribution of cytokine+ cells (percentage of gated cell subset) for each individual. Significant differences between groups, identified at p<0.05 by unpaired Student’s t-test, are underscored by connecting lines.
(TIF)
