# Supplementary figures and images for: Cynomolgus macaques naturally infected with Trypanosoma cruzi-I exhibit an overall mixed pro-inflammatory/modulated cytokine signature characteristic of human Chagas disease
Source: PLoS Negl Trop Dis. 2017 Feb 22;11(2):e0005233. doi: 10.1371/journal.pntd.0005233 (PMC5321273; doi:10.1371/journal.pntd.0005233)

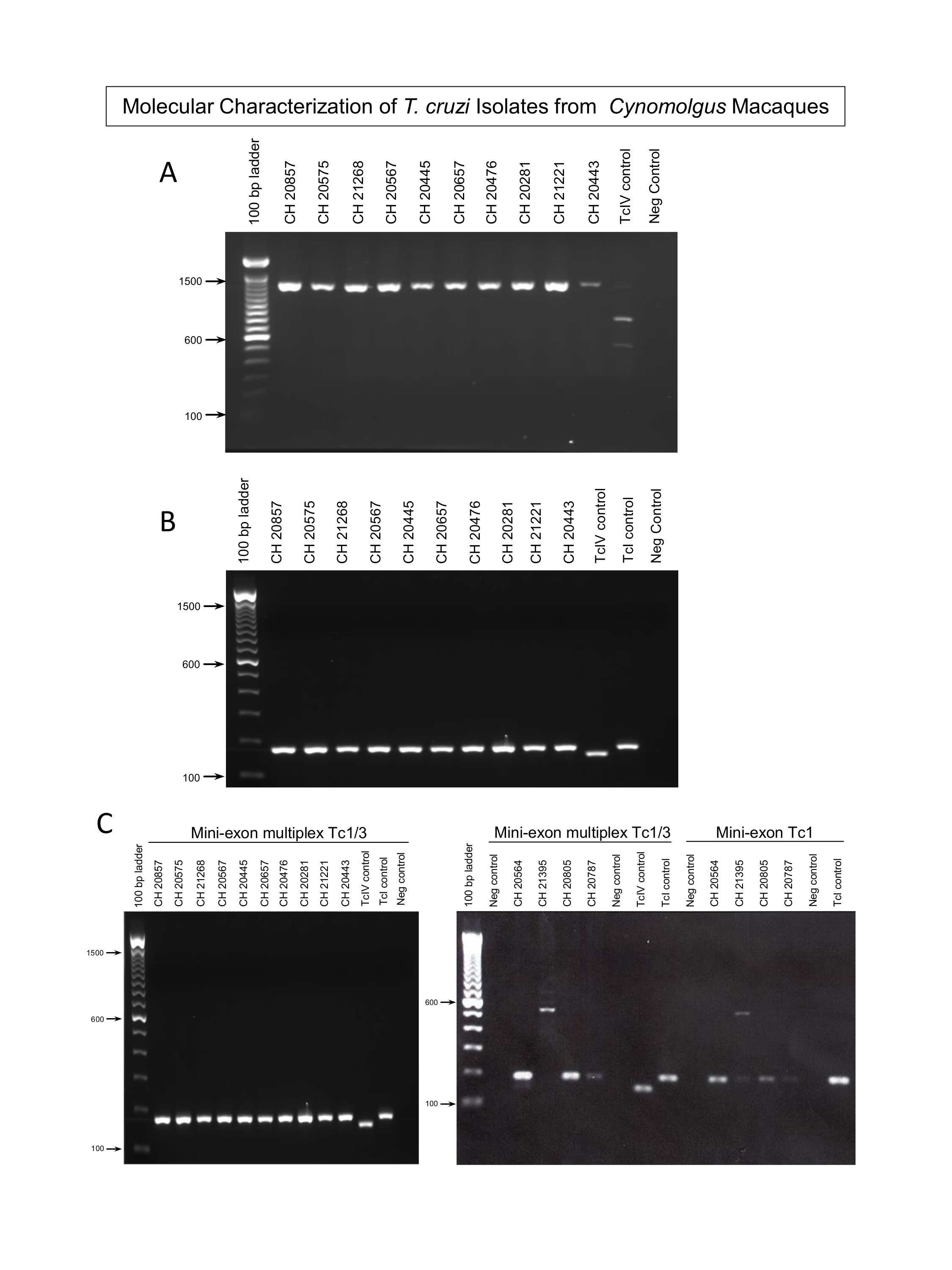

Supplement: S1 Fig — (A) PCR-RFLP characterization of the T. cruzi isolates from seropositive cynomolgus macaques (CH20857, CH20575, CH21268, CH20567, CH20445, CH20657, CH20476, CH20281, CH21221 and CH20443) using the GPI locus digested with the BstEII restriction enzyme as described in Material and Methods. (B) Characterization of the T. cruzi isolates from seropositive cynomolgus macaques (CH20857, CH20575, CH21268, CH20567, CH20445, CH20657, CH20476, CH20281, CH21221 and CH20443) using length variation at the 18S ribosomal RNA locus. (C) Characterization of the T. cruzi isolates from seropositive cynomolgus macaques (CH20857, CH20575, CH21268, CH20567, CH20445, CH20657, CH20476, CH20281, CH21221, CH20443, CH20564, CH21395, CH20805 and CH20787) using the combination of mini-exon primers Tc1, Tc3 and ME, previously described by Fernandes et al. [20]. Reference TcI and TcIV T. cruzi strains isolated from infected baboons from SNPRC and negative controls were included in the PCR batches. (TIF) [file pntd.0005233.s002.tif]

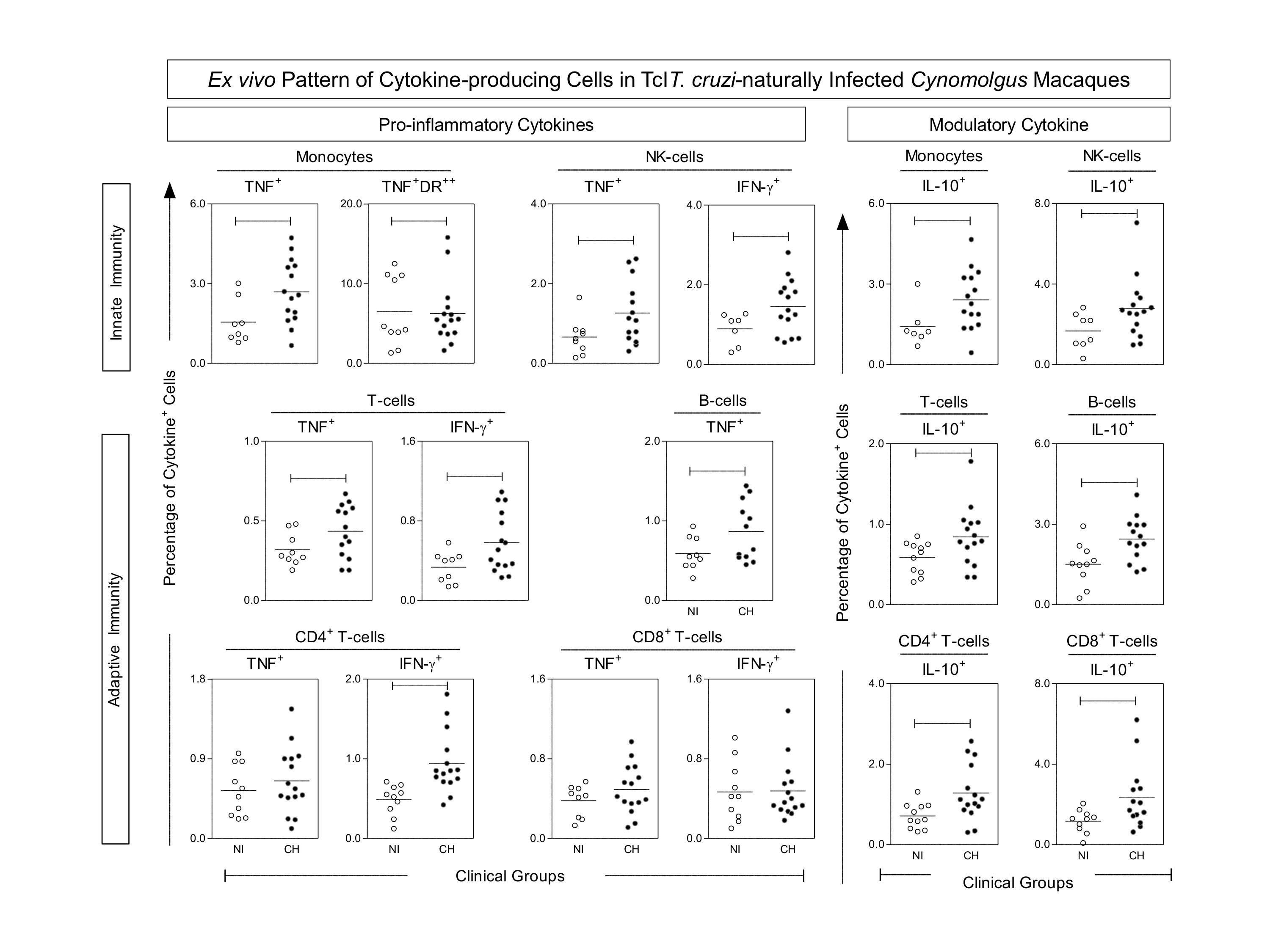

Supplement: S2 Fig — (A) Pro-inflammatory and (B) modulatory cytokine-producing cells in Chagas disease Cynomolgus hosts (CH, black circles) and non-infected controls (NI, white circles). The results are expressed as mean values and scattering distribution of cytokine+ cells (percentage of gated cell subset) for each individual. Significant differences between groups, identified at p<0.05 by unpaired Student’s t-test, are underscored by connecting lines. (TIF) [file pntd.0005233.s003.tif]
